# Predictors of Persistent Post-Traumatic Stress Symptoms After 2023 Maras Earthquake: The Impact of Gender, Loss, and Coping Strategies

**DOI:** 10.1007/s11126-025-10178-y

**Published:** 2025-06-24

**Authors:** Hasan Bakay, Beyza Isik, Sakir Gica, Mehmet Ak

**Affiliations:** Department of Psychiatry, Faculty of Medicine, Necmettin Erbakan University, Yunus Emre Mah., Beyşehir Cad. No:281,Meram, 42090 Konya, Türkiye

**Keywords:** Coping strategies, Earthquake, Loss, Psychological well-being, PTSD

## Abstract

The objective of this study was to ascertain the prevalence of trauma symptoms in individuals residing within the seismic region 18 months after the February 6th 2023, Maras earthquake, and to examine the factors contributing to these symptoms. The study included 339 participants who experienced the earthquake. The participants were administered sociodemographic data form, Traumatic Stress Symptom Checklist (TSSC), Coping with Earthquake Stress Scale (CESS), and Psychological Well-Being Scale (PWBS). According to the TSSC, 20.1% of the 339 participants were determined to have probable PTSD (pPTSD). In the pPTSD group, rate of destruction in the home, rate of women, rate of loss of life in relatives, rate of property loss, and rate of receiving psychiatric support were significantly higher. The pPTSD group demonstrated significantly lower positive reappraisal, seeking social support, and PWBS scores compared to the non-PTSD group. TSSC scores were negatively correlated with sub-scores of the CESS and PWBS among all participants. Regression analyses revealed that the presence of long-term pPTSD was predicted with being female, loss of life in relatives, property loss, and the need for psychiatric support. Additionally, positive reappraisal and seeking social support coping mechanisms were shown to decrease the risk of developing pPTSD. Current research suggest that traumatic symptoms may persist long after major natural disasters. Consequently, the provision of psychological support services, the enhancement of social support networks, and the dissemination of stress management methods following disasters such as earthquakes should be sustained over an extended period in high-risk regions. It is anticipated that the findings of this study will serve as a guide for researchers, clinicians, and policy-makers, facilitating the development of effective strategies for the management of post-disaster mental health needs.

## Introduction

On February 6, 2023, earthquakes measuring 7.6 and 7.7 on the Richter scale, centered in Maras, affected a large region covering 11 surrounding provinces. More than 11,000 aftershocks were recorded within a month of these massive earthquakes. More than 13 million people were affected by this earthquake, one of the biggest disasters in Turkey. According to official figures, there were more than 50,000 deaths and more than 100,000 injuries. Thousands of buildings collapsed or became unusable. Thousands of survivors lost their homes, loved ones or many of their possessions [1, 2]. In a meta-analysis examining the effects of different natural disasters on mental health, it was suggested that the effects of natural disasters on mental health decrease as time passes [3]. However, compared to other natural disasters, earthquakes can leave more permanent and chronic effects on human mental health due to reasons such as their sudden occurrence and aftershocks that can occur suddenly [4, 5]. Failures in social services that can be implemented after earthquakes with great destructive effects can further deepen this negative psychological impact [5].

It is known that exposure to a natural disaster can cause symptoms such as distress, fear, anxiety, depression and recurrent intrusive thoughts. These symptoms occur at levels that affect quality of life and functionality in many people [6, 7]. Although these disasters can lead to a wide range of psychiatric problems, posttraumatic stress disorder (PTSD) is a psychiatric disorder that is relatively more associated with such situations [8]. Gao et al. (2021) investigated the prevalence of psychiatric disorders long after the 2013 Lushan earthquake in earthquake-affected areas. As a result, they found that the prevalence of trauma and stress-related disorders was significantly higher in areas severely affected by the earthquake [9]. PTSD is a psychiatric disorder characterized by re-experiencing, avoidance and hyperarousal symptoms 1 month after a life-threatening event. This disorder can reach a frequency that can seriously affect public health after natural disasters such as earthquakes. There are reports of prevalence rates ranging from 4.10 to 67.07% in adults after earthquakes [10].

Coping strategies used by individuals against traumatic events may play a decisive role in the development of PTSD. It has been suggested that some individuals develop avoidant strategies (e.g. denial, suppression, anger venting) instead of confronting the trauma, and this leads to the inability to regulate negative emotions [11]. Especially after major traumas such as earthquakes, it has been claimed that trauma-oriented, maladaptive coping strategies such as avoidance and emotional suppression may increase the risk of PTSD, while adaptive coping strategies related to future-oriented, rebuilding a sense of trust and hope may reduce the risk of PTSD [5, 12–14]. In the light of the existing literature, it is of great importance to investigate the predictive effects of coping strategies on the emergence of PTSD in earthquake victims.

Numerous studies conducted in the aftermath of earthquakes have identified a wide range of risk factors associated with the development of probable PTSD (pPTSD), including female gender, younger age, loss of loved ones, property damage, and low levels of social support [1, 8, 15–17]. While some findings remain inconsistent—particularly regarding age and marital status—there is consensus that maladaptive coping strategies contribute significantly to poor psychological outcomes [5, 18, 19]. Additionally, although adaptive coping mechanisms such as positive reappraisal and seeking social support have been associated with improved mental health and post-traumatic growth, their long-term predictive value for pPTSD and well-being remains underexplored [5, 20, 21]. These inconsistencies and gaps in the literature highlight the need for studies that examine both protective and risk factors for pPTSD in long-term post-disaster contexts.

While previous studies have explored pPTSD in the aftermath of earthquakes and its association with various demographic and psychosocial variables, few have investigated these relationships over an extended post-disaster period. Moreover, most prior research has focused on early trauma reactions or acute phases. Current study contributes to the existing literature by examining not only the long-term prevalence of pPTSD 18 months after the 2023 Maras earthquake but also the predictive role of adaptive coping strategies—specifically positive reappraisal and social support seeking—on both pPTSD and psychological well-being. This integrative approach may allow for a more comprehensive understanding of sustained mental health outcomes in earthquake-exposed populations.

## Method

### Participants and Procedure

The study included 339 individuals who experienced the earthquake centered in Maras on February 6, 2023 and still residing in that region 18 months after the earthquake. The inclusion criteria were to be between the ages of 18–65, to be at least primary school graduate, to volunteer to participate in the study, to reside in the region affected by the February 6, 2023 earthquake and to have experienced the earthquake on that date. Exclusion criteria were being outside the age range of 18–65 years, being illiterate, having limited or retarded mental capacity understandable by the interview, not completing the questionnaires completely or having a medical disorder that may prevent the completion of the questionnaires. Participants were selected through convenience sampling among individuals who continued to reside in the Maras region 18 months after the earthquake. One of the researchers (BI) travelled to the affected area and conducted face-to-face interviews with the participants. All assessments, including sociodemographic data and psychometric scales, were administered in person. Prior to participation, written informed consent was obtained from each individual in accordance with ethical guidelines. Traumatic Stress Symptom Checklist (TSSC), Coping with Earthquake Stress Scale (CESS) and Psychological Well-Being Scale (PWBS) were administered to all participants along with sociodemographic data form. The PTSD subscale of the TSSC was shown to predict the diagnosis of PTSD with 81% sensitivity and specificity [22]. According to the TSSC, all participants were divided into pPTSD (*n* = 68, 20.1%) and non-PTSD (*n* = 271, 79.9%) and these two groups were compared. The power of the study was calculated as 0.90 (α-error 0.05, total sample size: 330 and effect size 0.35) using G*Power software (V3.1.9.2) [23].

### Measures

#### Sociodemographic Data Form

A personal information form was utilized for the purpose of collecting demographic information. This form was developed by the research team and inquired about the age, gender, marital status and education level of the participants. In addition, some information about whether the participants experienced loss of life and property in the earthquake, whether they had a psychiatric or physical illness before the earthquake, and whether they felt the need for psychiatric support after the earthquake were also questioned.

#### Traumatic Stress Symptom Checklist (TSSC)

TSSC is a 4-point Likert-type scale consisting of 23 questions in which individuals self-assess themselves in the last month. Each question has a score between 0 and 3 and the total scale score is obtained by summing the item scores. The first 17 items assess PTSD symptoms as specified in the Diagnostic and Statistical Manual of Mental Disorders-IV and the last six items assess depressive symptoms. A total score of 25 or higher on the 17 PTSD-related items indicates possible PTSD. Its sensitivity and specificity for PTSD have been reported as 81% [22]. The internal reliability of the TSSC was found to be excellent (Cronbach’s alpha = 0.91).

#### Coping with Earthquake Stress Scale (CESS)

This scale developed by Deniz Yöndem and Eren (2008) measures the coping strategies of individuals with earthquake stress. It consists of 16 questions in total and has 3 subscales. These subscales consist of religious coping, positive reappraisal and seeking social support. A high score indicates that the individual uses that coping strategy more. In the validity and reliability study of the scale, Cronbach’s α internal consistency coefficient was found to be 0.85, 0.69 and 0.74 for each subscale respectively [24].

#### Psychological Well-Being Scale (PWBS)

This scale, developed by Diener et al. (2009), consists of 8 seven-point Likert-type items, including positive relationships, sense of competence, having a meaningful and purposeful life, and being hopeful about the future. It is scored from strongly disagree (1) to strongly agree (7) and the scale can be scored between 8 and 56. A high score indicates that psychological well-being is high and in good condition [25]. In the validity and reliability study conducted in Turkey, Cronbach’s α coefficient was found to be 0.87 [26].

### Data Analysis

The statistical analyses were conducted utilizing the SPSS package (IBM SPSS Statistics for Windows, Version 22.0). Initially, the Kolmogorov-Smirnov and Shapiro-Wilk tests were employed to ascertain the normality of the distribution of the descriptive variables. Subsequent to this, the chi-square test was used to compare categorical variables between the groups. Finally, the Mann-Whitney U test was employed to compare the numerical data of both groups due to the non-normal distribution of the data. Spearman’s test was used to analyze the correlations between sociodemographic characteristics, psychometric test scores and complement levels within the patient group. Logistic regression analysis was also performed to determine the factors affecting the trauma level of the participants and the data were evaluated at a 95% confidence interval and a significance level of *p* ≤ 0.05.

## Results

### Sociodemographic Characteristics

Although it has been 18 months since the earthquake, 20.1% (*n* = 68) of the participants described intense PTSD symptoms (pPTSD). pPTSD and non-PTSD groups differed statistically in terms of gender, loss of relatives, loss of property, and damage to the house. The proportion of women in the pPTSD group was significantly higher (64.7% vs. 46.1%, *p* = 0.006), as were the rates of loss of life in relatives (52.9% vs. 33.6%, *p* = 0.003), loss of property (44.1% vs. 22.9%, *p* < 0.001) and severe damage to home (25% vs. 11.1%, *p* = 0.003). There was no significant difference between the two groups in terms of age, marital status, educational status and income. Please see Table 1 for detailed information.

**Table 1:** Comparison of sociodemographic features of pPTSD and non-PTSD groups (Mean ± SD)

		Group		Z / X^2^	*p*
		non-PTSD (*n* = 271)	pPTSD (*n* = 68)		
Age (median)		34.51 ± 12.9 (34)	34.85 ± 12.08 (34.5)	-0.539	0.590
Gender	Female Male	125 (46.1%) 146 (53.9%)	44 (64.7%) 24 (35.3%)	7.507	**0.006**
Year of education (median)		14.67 ± 2.83 (15)	14.79 ± 2.84 (16)	-0.901	0.368
Marital status	Married Single Widowed Divorced	144 (53.1%) 117 (43.2%) 3 (1.1%) 7 (2.6%)	35 (51.5%) 29 (42.6%) 2 (2.9%) 2 (2.9%)	1.298	0.730
Loss of life in relatives	Yes	91 (33.6%)	36 (52.9%)	8.698	**0.003**
Loss of property	Yes	62 (22.9%)	30 (44.1%)	12.402	**< 0.001**
Participation in rescue operations	Yes	76 (28%)	22 (32.4%)	0.491	0.483
Occupational status	Officer Housewife Student Other	120 (44.3%) 13 (4.8%) 98 (36.2%) 40 (14.8%)	34 (50%) 1 (1.5%) 21 (30.9%) 12 (17.6%)	2.575	0.462
Damage status of the home after the earthquake	Intact or slightly damaged Heavily damaged or demolished	241 (88.9%) 30 (11.1%)	51 (75%) 17 (25%)	8.883	**0.003**
Post-earthquake residence	Previously inhabited house Shelter or another place	210 (77.5%) 61 (22.5%)	47 (69.1%) 21 (30.9)	2.078	0.149
Income	Less than 17,000 TL 17,000–30,000 TL 30,000–50,000 TL 50,000–100,000 TL 100,000 TL and above	81 (29.9%) 41 (15.1%) 75 (27.7%) 69 (25.5%) 5 (1.8%)	20 (29.4%) 8 (11.8%) 22 (32.4%) 16 (23.5%) 2 (2.9%)	1.243	0.871
Presence of psychiatric disorder before the earthquake	Yes	19 (7%)	4 (5.9%)	0.110	0.741

*p ≤ 0.05, Chi-square and Mann-Whitney U tests were used, PTSD: post-traumatic stress disorder, pPTSD: probable post-traumatic stress disorder, SD: standard deviation

### Comparison of Psychometric Tests of Groups

When the two groups were compared in terms of coping strategies, CESS-Positive reappraisal and CESS-Seeking Social Support scores were significantly lower in the pPTSD group (*p* < 0.001, *p* = 0.018, respectively). Similarly, PWBS scores were also lower in the pPTSD group (*p* = 0.002). When CESS-Religious coping scores were compared, no significant difference was found between the two groups. Comparison of psychometric tests of groups was shown in Table 2.

**Table 2:** Comparison of psychometric tests of groups (Mean ± SD)

	Group		Z	*p*
	non-PTSD (*n* = 271)	pPTSD (*n* = 68)		
CESS-Religious coping (median) CESS-Positive reappraisal (median) CESS-Seeking Social Support (median) PWBS (median)	16.42 ± 3.29 (17) 18.89 ± 3.73 (19) 12.84 ± 2.64 (13) 42.33 ± 10.15 (45)	15.51 ± 3.43 (16) 16.54 ± 4.22 (16.5) 11.82 ± 3.08 (12) 37.66 ± 11.95 (39)	-1.866 -4.129 -2.373 -3.073	0.062 **< 0.001** **0.018** **0.002**

*p ≤ 0.05, Mann-Whitney U test was performed. PTSD: post-traumatic stress disorder, pPTSD: probable post-traumatic stress disorder, CESS: Coping with Earthquake Stress Scale, PWBS: Psychological Well-Being Scale, SD: standard deviation

### Correlations Between Sociodemographic Features and Psychometric Test Scores in Groups

A strong positive correlation was shown between age and CESS-Religious coping, CESS-Positive reappraisal and PWBS scores in all groups (*p* < 0.001, *p* = 0.006 and *p* = 0.001, respectively). It was also observed that as the TSSC-PTSD scores of the participants increased, their TSSC-Depression scores increased (*p* < 0.001). Both TSSC-PTSD and TSSC-Depression scores showed strong negative correlations with all coping strategy scores and psychological well-being scores. Finally, it was statistically strongly shown that as the participants’ coping scores (religious, positive reappraisal and seeking social support) increased, their psychological well-being scores also increased (*p* = 0.003, *p* < 0.001 and *p* < 0.001, respectively). In the pPTSD group, there was no correlation between TSSC-PTSD scores and coping scores, but a strong negative correlation was observed between TSSC-Depression scores and all coping strategy scores. In addition, there was no correlation between coping strategy scores other than CESS-Positive reappraisal and PWBS scores in the pPTSD group. See Table 3 for detailed information.

**Table 3:** Correlations between sociodemographic features and psychometric test scores in groups

Non-PTSD (*n* = 271)	Age	Year of education	TSSC-PTSD	TSSC-Depression	CESS-Religious coping	CESS-Positive reappraisal	CESS-Seeking Social Support	PWBS
*Age*	1							
Year of education	0.30**	1						
TSSC-PTSD	− 0.13*	− 0.05	1					
TSSC-Depression	− 0.07	− 0.03	0.74**	1				
CESS-Religious coping	0.39**	− 0.02	− 0.07	− 0.12	1			
CESS-Positive reappraisal	0.19**	0.07	− 0.18**	− 0.21**	0.42**	1		
CESS-Seeking Social Support	− 0.07	0.04	− 0.09	− 0.16**	0.04	0.14*	1	
PWBS	0.16**	0.09	− 0.31**	− 0.30**	0.14*	0.38**	0.20**	1
*pPTSD (n = 68)*								
Age	1							
Year of education	0.30**	1						
TSSC-PTSD	− 0.14	− 0.10	1					
TSSC-Depression	− 0.07	− 0.13	0.45**	1				
CESS-Religious coping	0.06	− 0.36**	0.05	− 0.12	1			
CESS-Positive reappraisal	0.08	− 0.11	0.19	− 0.21**	0.42**	1		
CESS-Seeking Social Support	0.14	0.04	0.06	− 0.16**	0.03	0.26*	1	
PWBS	0.32**	0.10	− 0.05	− 0.30**	0.14	0.26*	0.20	1
*All participants (n = 339)*								
Age	1							
Year of education	0.28**	1						
TSSC-PTSD	− 0.07	− 0.01	1					
TSSC-Depression	− 0.04	− 0.01	0.81**	1				
CESS-Religious coping	0.32**	− 0.09	− 0.12*	− 0.13*	1			
CESS-Positive reappraisal	0.15**	0.02	− 0.26**	− 0.28**	0.43**	1		
CESS-Seeking Social Support	− 0.03	0.03	− 0.14**	− 0.18**	0.05	0.19**	1	
PWBS	0.19**	0.07	− 0.31**	− 0.32**	0.16**	0.37**	0.22**	1

^*^p < 0.05, ^**^p < 0.01, Spearman’s correlation test was performed. PTSD: post-traumatic stress disorder, pPTSD: probable post-traumatic stress disorder, TSSC: Traumatic Stress Symptom Checklist, CESS: Coping with Earthquake Stress Scale, PWBS: Psychological Well-Being Scale

### The Effect of Sociodemographic Features and Psychometric Tests on Presence of pPTSD

Logistic regression analyses revealed that being female (B = 0.745, *p* = 0.02), experiencing loss of relatives (B = 0.608, *p* = 0.048), experiencing property loss (B = 0.817, *p* = 0.01), feeling the need for psychiatric support (B = 0.951, *p* = 0.002) predicted the persistent PTSD symptoms as long as 18 months after the earthquake. Coping strategies such as seeking social support and positive reappraisal were shown to play a protective role against the presence of pPTSD 18 months after the earthquake (B=-0.115, *p* = 0.03 and B=-0.122, *p* = 0.006, respectively). Table 4 demonstrates the examination of sociodemographic features and psychometric tests that are effective on the presence of pPTSD.

**Table 4:** Examination of sociodemographic features and psychometric tests that are effective on the presence of pPTSD by logistic regression analysis

Independent Variables	Presence of pPTSD				
	B	S.E.	EXP(B)	95.0% CI for β coefficient	*p*
Constant	0.330	1.138	1.390		0.772
Gender (1 = Female)	0.745	0.319	2.107	1.127–3.941	**0.02**
Loss of life in relatives (1 = Yes)	0.608	0.310	1.837	1.001–3.369	**0.048**
Loss of property (1 = Yes)	0.817	0.319	2.264	1.211–4.234	**0.01**
Need for psychiatric support (1 = Yes)	0.951	0.308	2.588	1.414–4.738	**0.002**
CESS-Religious coping	-0.02	0.05	0.984	0.893–1.085	0.75
CESS-Positive reappraisal	-0.115	0.042	0.891	0.821–0.967	**0.006**
CESS-Seeking Social Support	-0.122	0.056	0.885	0.793–0.988	**0.03**

Bold = *p* < 0.05, Logistic regression analyses test was performed. Cox & Snell R Square: 0.158, Nagelkerke R Square: 0.250, *p* ≤ 0.001. pPTSD: probable post-traumatic stress disorder, CESS: Coping with Earthquake Stress Scale, CI: confidence interval

## Discussion

The present study evaluated the prevalence of pPTSD and its associated factors 18 months after the 2023 Maras earthquake. The analysis revealed that being female, experiencing the loss of a relative, suffering property damage, and seeking psychiatric support were significantly associated with the presence of pPTSD. Furthermore, lower levels of positive reappraisal, seeking social support, and psychological well-being were observed among participants with pPTSD. While these risk factors increased the likelihood of pPTSD, adaptive coping strategies such as positive reappraisal and seeking social support played a protective role against the presence of pPTSD 18 months after the earthquake.

Regarding the frequency of pPTSD in the early post-earthquake period, literature show that pPTSD rates among earthquake-exposed individuals are around 50% [1, 15, 16]. A recent meta-analysis reported that 22.6% of 8868 earthquake victims were affected by PTSD [18]. In another more comprehensive meta-analysis including 46 studies, it was shown that PTSD rates were 28.76% within 9 months after the earthquake and PTSD diagnosis rates were 19.48% after 9 months [17]. Reinhardt et al. reported that the prevalence of PTSD was 18.6% 8 years after the 2008 Wenchuan earthquake [27]. In the light of the current literature, it can be said that PTSD rates tend to decrease gradually as the time after the earthquake prolongs. Especially after a period of one year, these rates plateau at a level of 20%. The rate of 20.1%, which we have determined in our study regarding pPTSD rates, is consistent with the current literature.

Regarding the relationship between PTSD and demographic data, in a study conducted in the early period after the Maras earthquake, younger age, female gender, being single, having a damaged house, loss of a close or loved one and low social support were found to be associated with PTSD [1, 15]. Similar to the general population, studies conducted on the causes of PTSD in women reported that being older, being injured, having a low level of education, having a severely damaged home, and having experienced loss of property and relatives were associated with PTSD [28, 29]. It has been claimed that women having stronger family ties and being more sensitive to the loss of relatives and being young may create susceptibility to PTSD through lack of life experience [1]. In other studies, older age, female gender, being single, being in an ethnic minority group, death in family members, damaged house, and lack of income were identified as risk factors for PTSD symptoms [16, 20]. In a meta-analysis of 2008 Wenchuan earthquake-related studies, it was demonstrated that the following factors enhance the likelihood of PTSD: being older, married, having a poor level of education, being female, having loss of loved one, and having little social support [8]. Although there is much evidence that home damage and property loss are associated with PTSD, Nobakht et al. (2019) pointed out that there are studies in the literature that suggest otherwise [30]. It has been reported that there is no relationship between having a psychiatric disorder in the past and the presence of post-earthquake PTSD [1, 31]. In our study, no such relationship was shown in line with these data. However, there are reports in the literature that pre-earthquake PTSD symptoms predict post-earthquake PTSD symptoms [32]. It may be possible to confirm this relationship with studies to be conducted on a larger sample.

As mentioned above, in the existing literature, there seems to be controversial data in terms of risk factors associated with PTSD after the earthquake, especially in terms of age and marital status. Although we showed strong positive correlations between age and coping scores and PWBS scores among all participants, we did not observe a significant correlation in the pPTSD group. Similarly, we did not observe a significant difference between the groups in terms of marital status. In terms of risk factors, female gender and bereavement can be defined as risk factors on which there is consensus. In addition, home damage, lack of social support, negative experiences after the earthquake, chronic medical diseases and low income have been emphasized as other risk factors [5, 18, 19, 33]. In our study, in line with the literature, the rates of women, those with loss of life in their relatives and those with severe damage to their homes were found to be significantly higher in the pPTSD group. In addition, we observed that property loss after the earthquake was also higher in the pPTSD group in the long term. Regression analyses showed that being female, experiencing loss of relatives, being exposed to property loss and the need for psychiatric support predicted the persistence of PTSD symptoms as long as 18 months after the earthquake.

Individual differences in coping styles may affect the positive or negative outcome of a traumatic experience. It has been shown that active coping styles, such as positive reappraisal, are associated with posttraumatic growth, whereas maladaptive coping styles are associated with PTSD symptoms [21]. Furthermore, it has been claimed that social behavior and social support can mitigate the devastating effects of post-earthquake PTSD [34]. One year after the 2009 L’Aquila earthquake in Italy, it was reported that maladaptive coping strategies such as denial, venting, behavioral disconnection and self-blame were more common in the group with PTSD symptoms, while adaptive coping strategies were more common in those without PTSD symptoms [20].

Religious coping is trying to overcome problems and find a way out with religious belief by exhibiting behaviors such as prayer and worship [35]. Positive reappraisal is associated with making positive meanings about the events experienced and thoughts that will contribute to personal development. It is generally associated with psychological well-being [24]. Social support is a factor that supports cognitive processes in overcoming the psychological effects of traumatic experiences [36]. In this context, it can be predicted that seeking social support may reduce the negative effects of trauma. In an early study conducted after the Maras earthquake, a negative correlation between the level of trauma and coping strategies, and a positive correlation between coping strategies and posttraumatic change in individuals exposed to the earthquake was shown [35]. Although religious coping has been widely associated with psychological resilience following natural disasters [37], our findings did not support a significant association between religious coping and the presence of probable PTSD in this sample. Cultural factors may influence how religious coping is utilized and experienced. In some cases, individuals may turn to religious practices as a form of avoidance rather than active problem-solving, which may limit its efficacy in mitigating PTSD symptoms [38]. Additionally, the timing of data collection could be relevant: while religious coping may offer short-term comfort in the immediate aftermath of trauma [39, 40], its long-term effects may diminish or become less predictive of psychological outcomes as individuals shift toward other coping mechanisms over time. One study reported that religious coping did not predict a reduction in PTSD symptoms after 1 year [41]. Another possible explanation is that, in regions where religious beliefs are deeply ingrained, religious coping may be used uniformly across affected populations regardless of symptom severity, making it less useful in distinguishing between those with and without probable PTSD. This might reduce variability and limit its predictive utility in regression models.

Löw et al. (2023) reported that passive coping strategies such as avoidance and emotional venting were more common in victims whose homes were damaged by earthquakes compared to those whose homes were not damaged. Another important finding of the authors is that the use of passive coping strategies is associated with higher PTSD symptoms. Using maladaptive coping methods such as stress avoidance and denial instead of seeking social support after the earthquake was found to be associated with PTSD [5]. Although seeking social support has been evaluated by some authors as an emotion-focused coping tool that does not bring a solution [42], social support-seeking behavior is an attitude aimed at increasing the adaptation of the person to the conditions he/she is in. In this context, it cannot be considered as a maladaptive attitude such as avoidance or denial. In addition, low social support is known to be a risk factor for PTSD [43]. Low perceived social support has also been found to be associated with PTSD [1]. Warner et al. (2015) suggested that social support provided by relatives in the early period after the earthquake is an important source of coping and mitigates the relationship between the loss of resources and the severity of posttraumatic stress reaction. They also reported that this support improved self-efficacy and that these positives persisted long after the earthquake [44]. In our study, we showed a statistically significant decrease in social support seeking behavior, which is one of the coping strategies, in the pPTSD group. Moreover, regression analyses revealed that the decrease in these behaviors predicted pPTSD even long after the earthquake. The finding that individuals who sought social support were less likely to develop pPTSD suggests that social support plays a protective role in long-term adjustment following disasters. As demonstrated in previous studies, interpersonal support has been shown to facilitate emotional regulation, reduce feelings of isolation, and strengthen resilience following trauma [45, 46]. In collectivist societies, such as Turkey, the act of seeking support is often culturally reinforced, thereby enhancing its perceived effectiveness. Consequently, the pursuit of social support emerges not only as a coping strategy but also as a pivotal factor in the attainment of sustained psychological recovery.

In the aftermath of the earthquake, it has been observed that not enough research has been conducted on the psychological well-being of communities. A recent study conducted after the Maras earthquake showed that psychological well-being was lower in individuals who were themselves or one of their nuclear family members affected by the earthquake, compared to individuals who had a distant relative affected by the earthquake or who were affected by the earthquake through the media. It was also reported that psychological well-being was lower in women among all participants [2]. In our study, PWBS scores were lower in the pPTSD group. Among all participants, we observed that PWBS scores increased with age. We showed a strong negative correlation between PTSD and depression scores, all coping strategy scores and PWBS scores. In addition, it was observed that as the coping scores of the participants increased, PWBS scores also increased. It is a fact that psychological well-being can be affected by many factors. Our findings provide important evidence that PTSD symptoms and coping mechanisms are associated with PWBS levels in individuals exposed to earthquake. However, further research on this relationship is needed. The association between adaptive coping strategies and higher psychological well-being observed in our study may be explained by the role these strategies play in promoting emotional regulation, self-efficacy, and meaning-making. Strategies such as positive reinterpretation and active problem-solving have been linked to better psychological functioning by reducing distress and enhancing a sense of control [47]. These mechanisms may serve to elucidate the protective influence of coping behaviors on overall well-being in the aftermath of trauma.

Our study has several limitations. First, the pPTSD group was defined according to the TSSC questionnaire. Although this questionnaire has reported high specificity and sensitivity rates for the diagnosis of PTSD compared to questionnaires used in other studies [22] the diagnosis of PTSD was not confirmed by face-to-face interviews. Second, the questionnaires were administered to the population in Maras province, the epicenter of the earthquake. The earthquake affected a much wider population than the epicenter. The data obtained from our study may not be generalizable to the entire population that experienced the earthquake. Finally, given that the present study concentrated on relatively contemporary and solution-focused coping mechanisms, the examination of maladaptive coping mechanisms was not included in the scope of the study.

Current study provides important evidence that traumatic symptoms may persist long after major natural disasters. In conclusion, provision of psychological support services, strengthening of social support networks and dissemination of stress management methods after major natural disasters such as earthquakes should be continued for a long time in high-risk areas. In addition to medical treatment, psychoeducation practices have been shown to be effective in reducing PTSD symptoms through coping strategies [48]. In this context, coping mechanisms may be an important intervention point in alleviating traumatic effects. It is thought that the findings obtained from this study will serve as a guide for researchers, clinicians and policy makers and facilitate the development of effective strategies for the management of post-disaster mental health needs.

## Data Availability

Data that support the findings are available from the corresponding author on reasonable demand.
